# Supplementary material for: Psychosocial work stressors and mental health in Ph.D. students in Germany—Evidence from two cross-sectional samples
Source: PLoS One. 2024 Dec 26;19(12):e0311610. doi: 10.1371/journal.pone.0311610 (PMC11670949; doi:10.1371/journal.pone.0311610)
Supplement: S1 Table — (PDF) [file pone.0311610.s001.pdf]

Table S1. Results from multiple linear regression analyses estimating associations between psychosocial stressors and mental health symptoms at T1 (n=159) and T2 (n=163) for only valid values.

	Model 1		Model 2		Model 3	
**T1**	**B[1]**	**95% CI[2]**	**B**	**95% CI[2]**	**B[1]**	**95% CI[2]**
Age	-.06	-.23;.11	-.03	-.19; .12	-.03	-.18; .12
Gender	.66	-1.59;2.91	.07	-1.98; 2.12	-.20	-2.18; 1.79
ER-ratio[3]			**5.60**	3.69; 7.52	**2.85**	.47; 5.22
Workload					.61	-1.39; 2.62
Boundary permeability					1.41	-.16; 2.98
Participation					-.99	-2.71; .73
Leader support					-1.351	-2.85; .15
Usability					-.512	-1.89; .87
**T2**						
Age	-.03	-.21; .15	-.05	-.20; .10	-.01	-.16; .13
Gender	1.51	-.77; 3.80	.77	-1.15; 2.70	.56	-1.31; 2.43
ER-ratio[3]			**6.39**	4.87; 7.92	**3.88**	1.78; 5.99
Workload					-.26	-1.97; 1.46
Boundary permeability					**2.50**	.98; 4.02
Participation					.19	-1.30; 1.67
Leader support					-1.08	-2.50; .33
Usability					-.58	-1.70; .55

In bold p-level <.05.

[1] Unstandardized regression coefficient.

[2] Confidence interval.

[3] Effort-reward ratio.
