# Supplementary material for: Psychosocial work stressors and mental health in Ph.D. students in Germany—Evidence from two cross-sectional samples
Source: PLoS One. 2024 Dec 26;19(12):e0311610. doi: 10.1371/journal.pone.0311610 (PMC11670949; doi:10.1371/journal.pone.0311610)
Supplement: S2 Table — (PDF) [file pone.0311610.s002.pdf]

Table S2. Results from multiple linear regression analyses estimating associations between psychosocial stressors and perceived stress at T1 (n=159) and T2 (n=163) for only valid values.

	**Model 1**		**Model 2**		**Model 3**	
**T1**	**B[1]**	**95% CI[2]**	**B[1]**	**95% CI[2]**	**B[1]**	**95% CI[2]**
Age	-.08	-.23; .08	-.05	-.19; .09	-.03	-.17; .10
Gender	.73	-1.31; 2.77	.17	-1.67; 2.01	-.12	-1.88; 1.64
ER-ratio[3]			**5.36**	3.64; 7.07	**3.66**	1.55; 5.77
Workload					-.49	-2.26; 1.29
Boundary permeability					1.00	-.39; 2.40
Participation					**-1.80**	-3.33; -.28
Leader support					-1.14	-2.47; .19
Usability					-.66	-1.88; .57
**T2**						
Age	-.04	-.21; .12	-.06	-.20; .08	-.02	-0.14; .11
Gender	1.71	-.38; 3.79	1.07	-.73; 2.87	.62	-1.01; 2.24
ER-ratio[3]			**5.47**	4.05; 6.90	**2.03**	0.20; 3.86
Workload					.15	-1.35; 1.64
Boundary permeability					**2.45**	1.13; 3.78
Participation					-.67	-1.96; .62
Leader support					**-1.77**	-3.00; -.53
Usability					-.63	-1.61; .35

In bold p-level <.05.

[1] Unstandardized regression coefficient.

[2] Confidence interval.

[3] Effort-reward ratio.
