# Supplementary material for: Psychosocial work stressors and mental health in Ph.D. students in Germany—Evidence from two cross-sectional samples
Source: PLoS One. 2024 Dec 26;19(12):e0311610. doi: 10.1371/journal.pone.0311610 (PMC11670949; doi:10.1371/journal.pone.0311610)
Supplement: Attachment — Submitted filename: Response to Reviewers.docx [file pone.0311610.s003.docx]

**Review Comments to the Author**

**Reviewer #1:**

Dear Authors,

Thank you for the opportunity to read your paper, which is dedicated to the critical subject of mental health and psychosocial work stressors among Ph.D. students at a German university.

The introduction is highly readable and has a good structure and flow. The background for undertaking the issue is clear and logical.

However, some minor and major issues should be addressed.

***Response:***

*Thank you very much for taking the time to read our manuscript and for your detailed and very constructive revision of our manuscript. We value your comments and believe that the following adjustments will substantially improve the manuscript.*

Minor issues:

1. Please add the percentage of Ph.D. student population in academia in the introduction

***Response:***

*Thank you for your insightful suggestion. We looked into the possibility of finding data to answer your question, but it proved challenging to obtain specific percentages of Ph.D. students among academic employees. In addition, these numbers can vary greatly for different countries. In Germany, for example, reports typically categorize staff into groups such as scientific employees or lecturers. While it is common for Ph.D. students to be included in the category of academic employees, this is not always the case, particularly for medical Ph.D. students. Unfortunately, we couldn't locate data that further distinguishes Ph.D. students within the scientific staff. However, we agree that such information would be valuable and important to have for future research as well.*

2. The diversification of Ph.D. students' faculty is low. Nevertheless, even though the highest rate of participants were students of Mathematical and Natural Sciences, there were also representatives of other faculties.

***Response:***

*Thank you for your comment. We agree with your statement that there is a low diversification. However, we wanted to include all gathered data to reach a broader range of experiences and to show results for all faculties. We have included a statement in our limitations section that this has to be kept in mind when viewing our results.*

3. Methods: What does it mean "a big university"? [line: 120] Please add the number of students in general and the number of Ph.D. students.

***Response:***

*Thank you again for this valuable comment. We have added the number of enrolled students and Ph.D. students to clarify what we meant by “big university”.*

*Please see line 126:*

*At this university, 30 763 students were enrolled in winter 2023/2024 and 3 562 were enrolled as Ph.D. students.*

4. Please add the description of measurement time in view of the academic year and Ph.D. students' responsibilities. The first measurement was conducted during the summer break, while the second between November and January. Yet, you report a higher imbalance during T1. How can you explain it?

***Response:***

*Thank you for your suggestion. We have already included a discussion of the potential influence of measurement time in the discussion, but we agree with you that we could have done this in a more detailed manner where we also include the discrepancy of T1 and T2.*

*Please see line 393:*

*In addition, we can already observe in our two samples that ER-ratios vary slightly for the two time points. As there is probably less interaction with students at T1 due to a summer break and thus less teaching responsibilities this may explain the higher imbalance of effort and reward when Ph.D. students may not receive the same amount of reward for their teaching from their students.*

5. Please add sample size calculation.

***Response:***

*We have added a sample size calculation.*

*Please see line 215:*

*For a statistical power of 0.8, a significance level of 0.05 with a medium effect size and eight predictors, at least 108 participants are required (33).*

6. Please describe how the common biases were controlled.

***Response:***

*Thank you for making us aware that we have lacked some descriptions of common bias in the limitations. We have added additional parts now, please see line 465:*

*In order to reduce the risk of selection bias, we have tried to address all enrolled Ph.D. students of the participating faculties at two time points with several reminder and by using two different study samples.*

*And line 484:*

*Third, as our study is only based on self-report measures, there may be a chance of common method bias (46). We have tried to reduce the risk of bias by using standardized and validated questionnaires that have been widely used in research.*

7. In the discussion, you only report the main associations, which are repeatable for T1 and T2. Please add to the discussion the differences between prediction models at T1 and T2 for Model 3. For example, leader support was associated with mental health at T1 but not at T2. In model 3 for associations of psychosocial stressors and perceived stress, participation and leader support were not significantly related to perceived stress at T2 (unlike at T1). Please explore it in the discussion.

***Response:***

*Thank you very much for bringing to our attention that we have missed to discuss these discrepancies between the two samples in more detail. We have added now the following parts in the discussion.*

*Please see line 396:*

*In addition to this, it can be observed that leader support is only significantly associated with mental health symptoms at T1, during the summer break, but not at T2. Thus, it can be speculated that leader support may only be relevant when additional support (i.e. from students) is lacking. For example,* *in a sample of university students it was shown that student support is an important factor which can reduce psychological distress (41). As components of leader support can be entailed in the reward component of the ERI model, this could also account for the lacking association between ER-ratio and mental health symptoms at T1.*

*Please further see line 423:*

*However, we could not observe similar findings for the study sample at T2 regarding participation. Explanations on this can only be speculative and need further clarification. For example, it may be possible that for participants during the second survey, having the additional teaching responsibility or having potential upcoming exams or deadlines may overshadow the potential effect of participation on stress perception. Another potential explanation would maybe offer the stage of dissertation project. For example, being at the beginning or end of studies could change how participation would impact stress perception.*

Major issues:

1. The repeated cross-sectional study design is a positive point of this study. However, the biggest issue is the sample representation. Less than 10% of P.D. students participated in this study, and the sample was from only one university. That undermines the results generalization. When you compare it to the Belgium sample, you compare it to the 15 times larger sample. Thus, it is expected that in a larger sample, the results would be flattened. So, unfortunately, the point of this comparison is missing. However, I agree with the Authors that this topic is vital and should be explored. Thus, I do not reject this paper based on a small sample size.

***Response:***

*Thank you for your valuable comment about our sample size and comparison to the Belgium sample. We agree with you that we should have mentioned the great difference in sample sizes of both samples to streamline the interpretation of the findings. As you pointed out correctly that it is expected to find flattened results in the Belgium sample, we have eliminated the relevance of this part of the paper by only considering it in the discussion section, as this goes also in line with recommendations from reviewer two. In regard of the sample size of our study, we have added a sample size calculation. We have also mentioned in the limitation section that our response rate was rather low, and that this may go along with selection bias.*

2. In Table 1 and Table 2, a significance of difference test with effect size should be added.

***Response:***

*Thank you very much for suggesting calculating a significance of difference test for our tables. With regard to the comment 1 and following your recommendations, we have excluded Table 2 from the manuscript and thus have only added t-tests and chi-square tests for Table 1.*

*Please see Table 1 for changes and also line 242:*

*The two samples showed significant differences in terms of effort, reward, workload, boundary permeability and leader support with weak effect sizes.*

3. Also, in my opinion, the year of study is missing, and it could be an essential variable. Please add to it to the models, if you have such a possibility.

***Response:***

*Thank you for this comment. We agree with you and would have also liked to include such a variable to our models. Unfortunately, we do not have the study year, but only one variable asking about the stage of the dissertation categorized into “in planning”, “in execution” or “in completion”. Following your line of thought we have repeated the analyses when dummies are included, but as no different results occurred or changes in the significance level were observed, we have decided to not include these post-hoc analyses in the manuscript.*

**Reviewer #2:**

Abstract: This is very good. I would also suggest including the percentage variance explained in the results with the p value. In the discussion section the first sentence needs greater clarity e.g. psychosocial stressors of PhD students and mental health symptoms are higher- but compared to what i.e. another sample, or is it that this particular sample has reported greater scores of mental health symptoms and perceived stress? It would also be helpful to give a specific direction for future research e.g. what types of methods should be used and how?

***Response:***

*Thank you very much for taking the time to read our manuscript and for commenting on how we can improve our manuscript. We have added the adjusted R^2^ in the abstract and also in the Tables in the results section. We have also added the p-value and B value for one example and have tried to make the first sentence of the discussion better understandable. We further included a sentence on how future research could improve and support our findings.*

*Please see line 36, 38 and 42:*

*For example, effort-reward-ratio showed a meaningful impact on mental health symptoms for the study sample at T2 (B=3.85; p<.05, adj. R^2^=0.288).*

*Both study samples showed high prevalence of mental health symptoms and effort-reward imbalance also in comparison to other research findings.*

*Future longitudinal studies could help to support our findings in terms of a causal stressor-strain association.*

Introduction

Lines 43 to 49: It's not clear why the authors begin by outlining mental health of academic staff. Academic staff and PhD students have very different roles and the narrative flow could be improved here. It would be better to remove the section on academics and focus on the key population (i.e. PhD students).

***Response:***

*Thank you very much for your thoughtful advice. We have started by framing our study topic in a broader context, namely that of academic staff. Ph.D. students are typically considered part of this group and are often not differentiated from the general scientific staff. Therefore, we believe it is important to first provide an overview of the general academic landscape, highlighting the unique position of Ph.D. students. Given their dual role, addressing their mental health in research is particularly crucial. To improve our narrative flow, we have included the following phrase, please see line 53:*

*…, as one occupational group within academia.*

Line 52. Should this be, 'every third person'?

***Response:***

*Yes, thank you. We have corrected the missing word.*

It would be helpful to comment on the wide literature, outlining poor student health, and to justify why PhD students might experience greater levels of stress compared to other students (undergraduate and postgraduate). For example, do PhD students have greater competing interests and commitments compared to other types of student? What causes stress in PhD students according to the qualitative evidence?

***Response:***

*Thank you very much for your considerate comment. We would like to clarify that our intention was to compare the situation of Ph.D. students with that of academic staff, rather than with students, as our focus is on exploring theoretical work stress models. This is why our introduction begins with a very short review of literature on the mental health of academics. We do believe that we have explained the special situation of Ph.D. students and their roles in academia in our introduction, which can result in different magnitudes of stressors, and which may also show the different commitments to students, for example. As we have mentioned in the introduction, there is also qualitative research, which could show for example, that effort and reward are important factors being reported by Ph.D. students.*

Line 98. Make sure you writing is explicit i.e. 'the DYNAMIK questionnaire entails five factors...'.

***Response:***

*Thank you for making us aware of our mistake. We have now explicitly mentioned the name of the questionnaire.*

Line 117- This is very clear, but I would also specify what the interventions are aimed at i.e. improving wellbeing/mental health.

***Response:***

*Thank you for this important comment, we have included the aim of the interventions.*

*Please see line 121:*

*Assessing psychosocial stressors with the DYNAMIK questionnaire for the first time among Ph.D. students may help to identify psychosocial risks in the workplace of Ph.D. students and thus shed new light on their working conditions and potential starting points for interventions improving mental health and perceived stress for Ph.D. students.*

Methods:

I would recommend splitting the participants section up from the study design. The participants section should focus on inclusion/exclusion criteria and how/where participants were recruited. You should not report how many participants you recruited here. This should be in the results section. The design section will be very short i.e. a note on the cross-sectional nature of the study, and why you completed two cross-sectional studies, as suggested in the title.

You should include a separate procedure section.

It needs to be clearer throughout the manuscript why two studies were conducted and what is meant by this.

***Response:***

*Thank you very much for your suggestion on how we can improve our method section. We have followed your suggestions and excluded the description of how many participants were recruited into the results section. Due to your comment, that the study design section would be very short, we have not made an extra paragraph, but have included another statement on the cross-sectional nature of the study. We have further clarified the importance and advantage of using two study samples in the section statistical analysis.*

*Please see line 197:*

*As only a small number of participants (n=52) filled in both questionnaires at T1 and T2, the two samples were treated as two cross-sectional samples in analyses. Using two different study samples can help to capture a broader range of experiences and stressors and can thus help to enhance generalizability of findings.*

*Please also see from line 218 onwards on the new paragraph about study participants in the results section.*

Statistical analysis:

Can you outline what the ER-ratio consists of. How was this captured?

***Response:***

*Please see the line 176 in the measure section for details on the ER-ratio. The ER-ratio consists of a ratio of the effort and reward scale and can thereby indicate whether an imbalance between these two components exists and in which magnitude. We have included another phrase to describe more in detail how we computed the ratio.*

*Please see line 181:*

*The ER-ratio was computed according to the formula by Siegrist et al. where effort is divided by reward and multiplied with a correction factor (32).*

Results:

Lines 219-229: This section should be in the discussion. In the results section you should only include a report of your results. However you can compare your results to other studies in the discussion.

***Response:***

*Thank you very much. We do agree with you and with reviewer 1 that this comparison does not improve our manuscript and therefore we have decided to eliminate this part and only discuss the findings in our discussion.*

Table 2 is not needed as it was not part of your hypotheses to focus on data from another study? This is not clearly discussed in the methods. The focus should be on your own results and reporting the predictors/association between stressors and mental health.

***Response:***

*Thank you again, as we agree with you and your line of thought we have as well excluded Table 2.*

One of the key limitations of your study is that psychosocial stressors will always predict mental health/distress/stress symptoms, because they are measuring the same underlying variables. I think by 'psychosocial stressors', you really mean 'psychosocial variables' (line 240). This makes more sense considering your variables.

***Response:***

*Thank you for your comment. We are not sure if we can completely follow your line of thought, but we would like to try to address this. By assessing both psychosocial stressors and perceived stress or mental health symptoms, we try to follow the stressor-strain framework. Of course, this would have been more adequately assessed within a longitudinal design, but we still believe that our research is important to report. The participants are exposed to several stressors during their working life, i.e. a high workload, and this results in different magnitudes of stress reactions when an individual evaluates such situations, contributing to stress perceptions. However, we do agree with you that it is a limitation of our study that we have assessed both stressors and strain from the same participants (i.e. common method bias), as these concepts are strongly associated with each other. Therefore, we have included a statement on this in our limitation section.*

*Please see line 485:*

*Third, as our study is only based on self-report measures, there may be a chance of common method bias (46). We have tried to reduce the risk of bias by using standardized and validated questionnaires that have been widely used in similar research.*

The variance explained by the model should be described in the results. This may be useful to identify what is important to report: https://statistics.laerd.com/spss-tutorials/multiple-regression-using-spss-statistics.php

***Response:***

*Thank you for making us aware that we have missed this. We have added the adjusted R^2^ in our results tables 2 and 3.*

Discussion:

I think a broader focus on the factors predicting/associated with stress are needed in the discussion. The variables explored are generally discussed well. Some sections could be more concise e.g. practical implications.

***Response:***

*Thank you very much for your suggestion. We do not fully understand, which factors you are referring to and would like to ask if you could give examples on how we can improve the discussion section and where you think a broader focus is needed. We have tried to be more concise in our implications and have shortened this section to findings relevant for our study, thank you for this suggestion.*

This paper is very interesting but there are several areas which need clarification.

***Response:***

*We hope and believe that we could improve our manuscript with your valuable comments.*
